# A computed tomography-based nomogram for neoadjuvant chemotherapy plus immunotherapy response prediction in patients with advanced esophageal squamous cell carcinoma

**DOI:** 10.3389/fonc.2024.1358947

**Published:** 2024-06-05

**Authors:** Wen-wen Guo, Chuanqinyuan Zhou, Dan Gao, Min Xu, Yan Gui, Hai-ying Zhou, Tian-wu Chen, Xiao-ming Zhang

**Affiliations:** ^1^ Medical Imaging Key Laboratory of Sichuan Province, and Department of Radiology, Affiliated Hospital of North Sichuan Medical College, Nanchong, Sichuan, China; ^2^ Department of Oncology, Affiliated Hospital of North Sichuan Medical College, Nanchong, Sichuan, China; ^3^ Department of Radiology, The Second Affiliated Hospital of Chongqing Medical University, Chongqing, China

**Keywords:** esophagus, squamous cell carcinoma, immunotherapy, chemotherapy, computed tomography, nomogram

## Abstract

**Objective:**

To develop a CT-based nomogram to predict the response of advanced esophageal squamous cell carcinoma (ESCC) to neoadjuvant chemotherapy plus immunotherapy.

**Methods:**

In this retrospective study, 158 consecutive patients with advanced ESCC receiving contrast-enhanced CT before neoadjuvant chemotherapy plus immunotherapy were randomized to a training cohort (TC, *n* = 121) and a validation cohort (VC, *n* = 37). Response to treatment was assessed with response evaluation criteria in solid tumors. Patients in the TC were divided into the responder (*n* = 69) and non-responder (*n* = 52) groups. For the TC, univariate analyses were performed to confirm factors associated with response prediction, and binary analyses were performed to identify independent variables to develop a nomogram. In both the TC and VC, the nomogram performance was assessed by area under the receiver operating characteristic curve (AUC), calibration slope, and decision curve analysis (DCA).

**Results:**

In the TC, univariate analysis showed that cT stage, cN stage, gross tumor volume, gross volume of all enlarged lymph nodes, and tumor length were associated with the response (all *P* < 0.05). Binary analysis demonstrated that cT stage, cN stage, and tumor length were independent predictors. The independent factors were imported into the R software to construct a nomogram, showing the discriminatory ability with an AUC of 0.813 (95% confidence interval: 0.735–0.890), and the calibration curve and DCA showed that the predictive ability of the nomogram was in good agreement with the actual observation.

**Conclusion:**

This study provides an accurate nomogram to predict the response of advanced ESCC to neoadjuvant chemotherapy plus immunotherapy.

## Introduction

Esophageal cancer is the seventh most common malignancy and the sixth most common cause of cancer-related deaths worldwide (1, 2). Esophageal squamous cell carcinoma (ESCC) is the predominant histological type (3). Surgery is the main element of treatments for resectable esophageal cancer (1), but the 3-, 5-, and 10-year survival rates were 35%, 25%, and 18%, respectively (4). Most patients with ESCC had already reached the locally advanced stage at the time of diagnosis because of subtle symptoms at the early stage, and their prognoses were usually unsatisfactory. Neoadjuvant therapy (radiotherapy, chemotherapy, or a combination) before surgery has the advantage of targeting micrometastases and increasing complete resection rates (5), and for locally advanced ESCC, neoadjuvant therapy has already become one of the standard treatment options.

Immunotherapy has been recognized as an exciting therapeutic strategy for the treatment of various types of cancer in recent years, which uses the patient’s own immune system to fight malignant cells by suppressing the immune checkpoint pathway (6, 7). After a long debate about whether the immune system can recognize and kill tumor cells specifically, a growing body of evidence indicates that immune cells do indeed play an important role in controlling tumor cells (8). An immune checkpoint inhibitor, such as programmed death 1 (PD-1)/programmed death-ligand 1 (PD-L1) inhibitors, rescues the antitumor effect of T cells by blocking the immune checkpoint pathway to fight cancer (9). Anti-PD-1 antibody plus chemotherapy may extend the survival time of ESCC patients and has already become the standard treatment (10). According to the results from randomized phase III trials, immune checkpoint inhibitors in combination with chemotherapy have been recommended as the first-line treatment instead of chemotherapy alone for patients with advanced esophageal cancer (11–15). Hence, neoadjuvant chemotherapy plus immunotherapy has become one of the most important treatment regimens for advanced ESCC. Preoperative neoadjuvant chemotherapy plus immunotherapy can further improve the survival rate and reduce the risk of distant metastasis and local recurrence for advanced ESCC patients. However, some patients may not benefit from it and may miss valuable surgical opportunities.

CT plays a key role in initial diagnosis, guidance of treatment, and subsequent follow-up for patients with esophageal cancer (16, 17). CT has been applied to predict PD-L1 and CD8^+^ TIL expression levels in ESCC patients and differentiate between immune checkpoint inhibitor-related pneumonitis and radiation pneumonitis for patients with unresected locally advanced stage non-small cell lung cancer (18, 19). To our knowledge, there exist no reports on the response prediction in patients with advanced ESCC who were treated with neoadjuvant chemotherapy plus immunotherapy. Therefore, our study aimed to develop and validate a CT-based nomogram to predict the response to neoadjuvant chemotherapy plus immunotherapy in patients with advanced ESCC, in order to assess the patients’ response to neoadjuvant chemotherapy plus immunotherapy based on pretherapeutic CT images and recommend neoadjuvant treatments to the “responders.”

## Materials and methods

### Patients

Our institutional ethics committee approved this study, and an informed consent was signed by each patient before partaking in this study.

We retrospectively collected and analyzed patients with advanced ESCC who received neoadjuvant chemotherapy plus immunotherapy in our hospital from January 2020 to September 2022. The inclusion criteria were as follows: 1) diagnosis of ESCC based on preoperative endoscopic pathological examination, 2) patients underwent CT before neoadjuvant therapy, and 3) patients’ clinical TNM stage was cT_3-4a_N_0-2_M_0_ as depicted on CT. According to the inclusion criteria, we collected 175 consecutive cases. The exclusion criteria were any of the following: 1) patients received any tumor-related treatments (e.g., chemotherapy or radiotherapy) before undergoing CT (*n* = 10), 2) patients had concomitant malignant tumors of another type (*n* = 2), or 3) the quality of the images was not good enough (*n* = 5). Therefore, 17 cases were excluded from our study, and 158 patients were ultimately included in the study. All participants were randomly assigned to a training cohort (TC, *n* = 121) and a validation cohort (VC, *n* = 37) with SPSS (version 25, Chicago, IL, USA) (20). All the patients underwent CT examinations before and after two cycles of neoadjuvant chemotherapy plus immunotherapy. The age, gender, and anatomic distribution of the tumor in the TC and VC are depicted in **Table 1**.

**Table 1:** Clinical and demographic data of all enrolled patients.

Variable	Training cohort	Validation cohort
Total number of patients (responder:non-responder)	121 (69:52)	37 (22:15)
Sex, male:female	93:28	28:9
Age, median (range) in years	66 (49–80)	67 (51–81)
Neoadjuvant regimen, *n* (%)		
Paclitaxel, cisplatin, and camrelizumab	12 (9.9%)	3 (8.1%)
Paclitaxel, carboplatin, and sintilimab	49 (40.5%)	14 (37.9%)
Docetaxel, carboplatin, and sintilimab	19 (15.7%)	5 (13.5%)
Paclitaxel, carboplatin, and pembrolizumab	6 (5.0%)	5 (13.5%)
Paclitaxel, cisplatin, and sintilimab	18 (14.9%)	6 (16.2%)
Docetaxel, cisplatin, and sintilimab	17 (14.0%)	4 (10.8%)
cT stage, *n* (%)		
cT_3_	77 (63.6%)	24 (64.9%)
cT_4_	44 (36.4%)	13 (35.1%)
cN stage, *n* (%)		
cN_0_	72 (59.5%)	22 (59.5%)
cN_1_	37 (30.6%)	13 (35.1%)
cN_2_	12 9.9%)	2 (5.4%)
Anatomic distribution, *n* (%)		
Upper thoracic segment	21 (17.4%)	6 (16.2%)
Middle thoracic segment	81 (66.9%)	21 (56.8%)
Lower thoracic segment	19 (15.7%)	10 (27.0%)
GTV (cm^3^)	20.54 (13.89, 28.08)	15.02 (9.02, 22.21)
GVALN (cm^3^)	0 (0, 3.64)	0 (0, 6.79)
Tumor length (cm)	5.42 (4.56, 6.69)	5.01 (3.75, 6.09)

Continuous values are expressed as median (25% quantile, 75% quantile).

GTV, gross tumor volume; GVALN, gross volume of all enlarged lymph nodes.

### Contrast-enhanced CT scans

All image data were acquired using a 64-section multidetector computed tomography (MDCT) (LightSpeed VCT, GE Medical Systems, USA). Patients were orally given 100 ml to 200 ml of water as a negative contrast agent before CT data collection. The CT examinations were performed in a supine position. First, the routine unenhanced scans were performed. Subsequently, a 70–100-ml contrast agent (Omnipaque, Iohexol, GE Healthcare, USA), calculated based on a ratio of 1.5 ml/kg body weight, was injected into an antecubital vein with a 20-G needle at the rate of 3.0 ml/s, and then, 20 ml of saline was rinsed with a pump syringe (Vistron CT Injection System, Medrad, USA). Twenty to 30 s after the contrast injection, contrast-enhanced CT data were obtained. The CT scanning parameters were as follows: peak voltage 120 kV, tube current 200 mA (using automatic exposure control), rotation time 0.5 s, collimation 64 × 0.6 mm, pitch 0.9, section thickness 5 mm, and matrix 512 × 512 mm. The CT scan covered the area from the neck to the middle of the left kidney. During one breath-hold with a full-held inspiration for 10–15 s, each examination was performed. Finally, CT data were transmitted directly to the General Electric Advantage Workstation 4.4 at the mediastinal window settings, and the window width was 400 HU with a window level of 40 HU.

### Treatment

All enrolled patients received two cycles of neoadjuvant chemotherapy plus immunotherapy. The concrete treatment drugs were as follows (21, 22): taxel (docetaxel or paclitaxel) and platinum doublet (cisplatin or carboplatin) plus immunotherapy (sintilimab, pembrolizumab, or camrelizumab). The details of the above chemotherapy were as follows: during each cycle of the 21-day duration, cisplatin or carboplatin (75 mg/m^2^) on day 1, docetaxel or paclitaxel (135 mg/m^2^) on days 1 and 8, and sintilimab, pembrolizumab, or camrelizumab (200 mg) on day 1 were administered intravenously. All patients were re-evaluated with a CT scan of the chest and upper abdomen approximately 4 to 6 weeks after neoadjuvant chemotherapy plus immunotherapy. The neoadjuvant regimens of the patients in the TC and VC are depicted in **Table 1**.

### Assessment of response

Due to the absence of the phenomenon of pseudoprogression in this study, tumor response was evaluated based on the Response Evaluation Criteria in Solid Tumors (RECIST) version 1.1 as follows (23): complete response (CR) was the disappearance of all target lesions; partial response (PR) was at minimum a 30% reduction in the sum of the longest target lesion diameters using the sum of the longest target lesion diameters at baseline as reference; progressive disease (PD) was at minimum a 20% increase in the sum of the longest target lesion diameters using the minimum sum of the longest target lesion diameters recorded since the start of treatment (rock bottom) as reference, or the appearance of at least one new lesion; and stable disease (SD) was neither PR nor PD. The patients with CR or PR according to RECIST (v.1.1) within two treatment cycles were considered “responders,” while those who suffered from PD or SD were considered “non-responders.” As reported (24), the application of iRECIST had no impact on response-related endpoints, compared to RECIST 1.1, and we used RECIST 1.1 to assess the tumoral treatment response.

### Pretherapeutic CT features assessment

**Figure 1:**
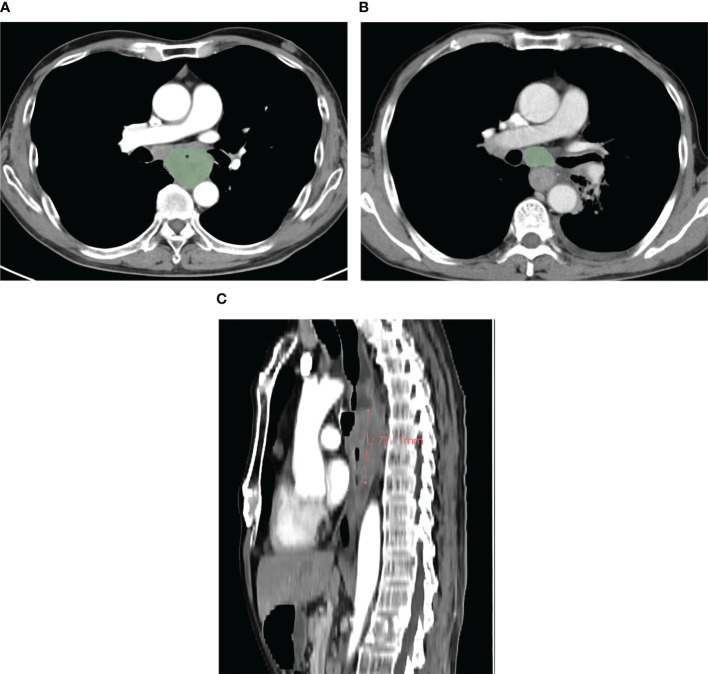
In a 59-year-old male patient with cT_4_N_1_M_0_ esophageal squamous cell carcinoma, the pretherapeutic contrast-enhanced CT scans show the gross tumor volume obtained by slice-by-slice manual delineation of the tumor **(A)**, and the gross tumor volume is 57.66 cm^3^. In a 64-year-old male patient with esophageal squamous cell carcinoma at cT_3_N_1_M_0_, the gross volume of the enlarged lymph nodes was obtained in a way similar to that of the tumor **(B)**, and the gross volume of all enlarged lymph nodes is 21.28 cm^3^. In a 59-year-old male patient with cT_4_N_2_M_0_ esophageal squamous cell carcinoma, the tumor length was obtained with the value of 7.11 cm **(C)**.

The pretherapeutic CT features of advanced ESCC were assessed on the 3D-slicer 4.11. The esophageal wall thickness that exceeded 5 mm on axial contrast-enhanced CT was considered abnormal thickness caused by the tumor (25). The pretherapeutic CT features included cT stage, cN stage, anatomic distribution, gross tumor volume (GTV), gross volume of all enlarged lymph nodes (GVALN), and tumor length. The shape of the esophageal tumor was manually outlined around the abnormal tissue on enhanced CT (**Figure 1**) independently by two radiologists (observer 1 with 3 years of expertise in radiology and observer 2 with 4 years of expertise in radiology), and then the above software automatically calculated the GTV. The volume of each enlarged lymph node was also obtained by the previous two radiologists in a way similar to the previous GTV, and GVALN was obtained by the sum of the volume of each enlarged lymph node. The tumor lengths were independently assessed by observers 1 and 2 on axial and sagittal contrast-enhanced CT images as follows: the upper and lower edges of the tumor on axial contrast-enhanced CT images were determined and marked, and then we measured the tumor length on sagittal contrast-enhanced CT images through the markers with the referring standards of double contrast barium examinations. The measurements of GTV, GVALN, and tumor length of ESCC independently by the above two radiologists were used to test the interobserver reproducibility. Observer 1 remeasured the GTV, GVALN, and tumor length of all target lesions 1 month later to test the intraobserver reproducibility. Before their measurements, a radiology professor with 25 years of radiology experience taught them how to assess quantitative CT tumor features randomly in 20 patients.

For assessing the pretherapeutic categorical CT features including cT stages, cN stages, and anatomic distribution, observer 1 discussed with observer 2 to reach a consensus. When observer 1 and observer 2 disagreed with each other, they could consult the above professor. The cT and cN stages were assessed using the pretherapeutic CT data based on the 8th edition of the American Joint Committee on Cancer (AJCC) staging of cancers of the esophagus and esophagogastric junction (26). For the cT stage, cT_3_ of the advanced tumor infiltrated the adventitia, and the cT_4_ tumor infiltrated adjacent structures. For the cN stage, the number of involved nodes determines the N stage: the N_0_ stage involves no node, the N_1_ stage involves one to two nodes, the N_2_ stage involves three to six regional nodes, and the N_3_ stage involves seven or more regional nodes. Based on the AJCC staging of cancers of the esophagus and esophagogastric junction, the short axis of nodes can be easily measured on CT, and the intrathoracic and abdominal lymph nodes >1 cm can be considered enlarged. All evaluations were performed without knowledge of the histological results.

### Statistical analysis

Statistical analyses were performed by SPSS (version 25, Chicago, IL, USA). According to the published study on pancreatitis using similar statistical analysis (27), univariate analysis was performed to identify the pretherapeutic CT features associated with the response prediction. Chi-squared test or Fisher’s exact test was used to compare categorical variables, and the independent-sample *t*-test or Mann–Whitney ***U*** test was used to compare continuous variables. Binary logistic analysis was performed for statistically significant CT features from the univariate analysis and showed no multicollinearity (27). A *P <*0.05 was considered statistically significant for all statistical tests.

### Nomogram development and validation

The R software (version 4.2.2) was used to develop and validate the nomogram. For the development of the nomogram, only independent predictive factors determined by binary logistic analysis were selected. In our nomogram, the regression coefficient of each independent predictive factor in binary logistic regression was proportionally transformed into a specific number on a scale of 0 to 100 points. Moreover, the accuracy of the predictive nomogram was assessed using the concordance index (C-index) and the calibration curves. The receiver-operating characteristic (ROC) curve and the largest area under the receiver-operating characteristic curve (AUC) were obtained with the optimal cutoff point in the nomogram. Additionally, we used decision curve analysis (DCA) to validate the clinical application value of our model because DCA is a novel algorithm for evaluating the net benefit value of a model under different thresholds (28).

## Results

### Response in patients

In the TC, 9 (7.4%), 60 (49.6%), 41 (33.9%), and 11 (9.1%) patients demonstrated CR, PR, PD, and SD, respectively. In the VC, 2 (5.4%), 20 (54.1%), 11 (29.7%), and 4 (10.8%) patients demonstrated CR, PR, PD, and SD, respectively. Hence, there were 69 responders and 52 non-responders in the TC and 22 responders and 15 non-responders in the VC.

### Intra- and interobserver measurement agreement of quantitative features

Intraobserver and interobserver agreements of GTV, GVALN, and tumor length are shown in **Table 2**. Both intra- and interobserver ICC values of the above measurements were >0.90 (*P* < 0.001 for all). Thus, the GTV, GVALN, and tumor length obtained by the first measurements from the first observer were used for the subsequent analyses.

**Table 2:** The intra- and interobserver agreements in the quantitative measurements.

Parameters	Intraobserver ICC values	Interobserver ICC values
GTV	0.986 (95% CI, 0.947–0.997)	0.989 (95% CI, 0.958–0.997)
GVALN	0.979 (95% CI, 0.904–0.995)	0.983 (95% CI, 0.920–0.996)
Tumor length	0.978 (95% CI, 0.911–0.995)	0.963 (95% CI, 0.857–0.990)

CI, confidence interval; ICC, interclass correlation coefficient; GTV, gross tumor volume; GVALN, gross volume of all enlarged lymph nodes.

### Univariate analysis of pretherapeutic CT and clinical features in the TC: correlation with response

The pretherapeutic CT and clinical features are listed in **Table 1**. According to our univariate analysis, the cT stage, cN stage, GTV, GVALN, and tumor length were associated with response in patients with ESCC after neoadjuvant chemotherapy plus immunotherapy (*P* < 0.05 for all). In detail, patients with lower cT stage, lower cN stage, lower GTV, lower GVALN, and lower tumor length were more likely to be responders (*P* = 0.001, < 0.001, = 0.001, = 0.002, and < 0.001, respectively). However, there were no statistically significant differences in gender, age, neoadjuvant regimen, and anatomic distribution between responders and non-responders (*P* = 0.376, 0.717, 0.617, and 0.094, respectively).

### Binary analysis of CT features with response in the TC

As for potential independent predictive factors for therapeutic response after neoadjuvant chemotherapy plus immunotherapy including cT stage, cN stage, GTV, GVALN, and tumor length, the binary logistic regression analysis was used for identifying the independent predictive factors. The results of the binary logistic regression analysis indicated that cT stage, cN stage, and tumor length were independent predictive factors in patients with advanced ESCC (*P* = 0.039, 0.001, and 0.002, respectively).

### CT-based nomogram for prediction of therapeutic response in the TC

**Figure 2:**
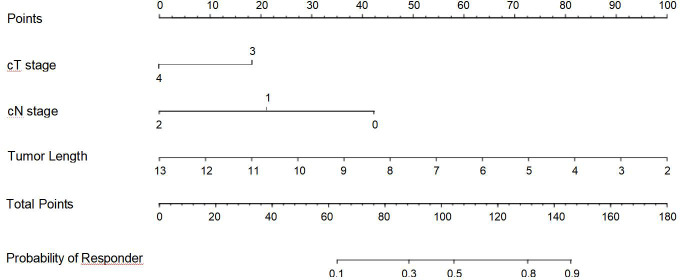
Nomogram for predicting the response of advanced esophageal squamous cell carcinoma after neoadjuvant chemotherapy plus immunotherapy.

Based on the above binary analysis in the TC, a nomogram was constructed to predict the response of patients with ESCC to neoadjuvant chemotherapy plus immunotherapy that incorporated the three independent predictive factors: cT stage, cN stage, and tumor length (**Figure 2**). A total score was calculated with the use of cT stage, cN stage, and tumor length which were reachable on CT. In our nomogram, the value of each variable was scored on the axis of the score scale. By adding each score, the total score could be easily calculated, and we were able to predict patients’ response after the neoadjuvant treatment by projecting the total score onto the total point scale.

### Performance of the CT-based nomogram in the TC and VC

**Figure 3:**
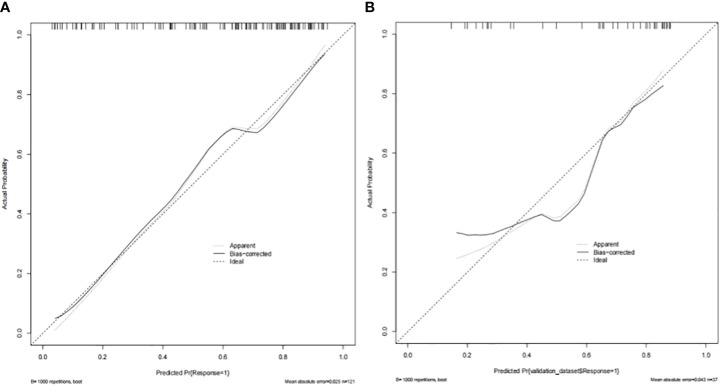
The nomogram’s calibration curves in the training cohort **(A)** and the validation cohort **(B)**.

**Figure 4:**
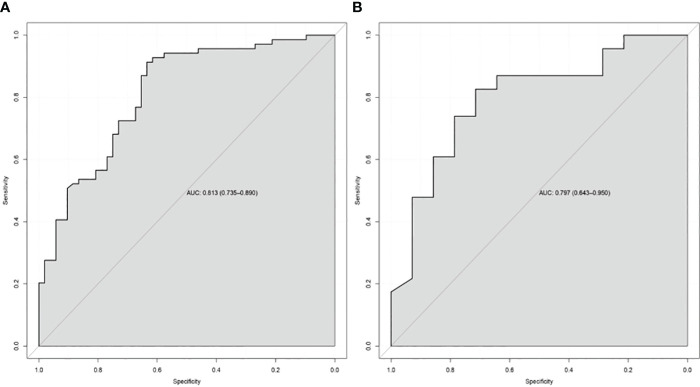
The receiver-operating characteristic curve of the nomogram of the training cohort **(A)** and the validation cohort **(B)**. AUC, the area under the receiver-operating characteristic curve.

**Figure 5:**
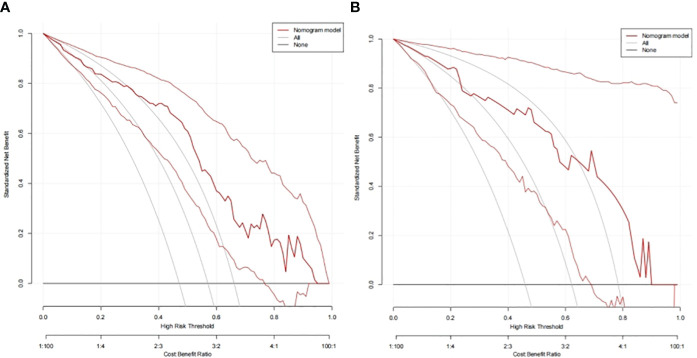
Decision curves of the nomogram to predict response in the training cohort **(A)** and the validation cohort **(B)**.

In the TC and VC, an individual calibration curve was used to confirm the accuracy of the nomogram based on the predictive nomogram, showing that the predicted values of our model coincided with the actual values (**Figure 3**). The AUC values of 0.813 [95% confidence interval (CI), 0.735–0.890] in the TC and of 0.797 (95%CI, 0.643–0.950) in the VC suggested excellent predictive power, respectively (**Figure 4**). DCA demonstrated good clinical application of this nomogram in the TC and VC (**Figure 5**).

## Discussion

Previous research studies (29, 30) have explored the treatment response to chemoradiotherapy in patients with esophageal cancer based on CT. Immunotherapy has become increasingly important in patients’ treatments, but there is no literature on the prediction of response to this therapy. The current study shows that cT stage, cN stage, and tumor length depicted on CT are independent predictive factors in patients with advanced ESCC who have received neoadjuvant chemotherapy plus immunotherapy for the first time. We investigated the feasibility of a nomogram developed with independent predictive factors to predict the response.

As shown in this study, the cT stage and cN stage of the tumor could be independent factors in predicting response to neoadjuvant chemotherapy plus immunotherapy. Previous studies manifested that the clinical stage could predict the response to neoadjuvant treatment (31–33). The higher the cT stage, the higher the probability of non-responder could be. This is due to the invasion of blood vessels, cancer cells entering the bloodstream and causing metastasis, which leads to adverse results. The lack of metastatic lymph nodes indicates that the disease is still at an early stage, suggesting a superior response. Qiao et al. reported that the pathologic CR is more likely to appear in patients without lymph node metastases (34). Several published papers have shown the appearance of metastatic lymph nodes is a major predictive factor contributing to high recurrence rate in esophageal cancer (35, 36).

This study revealed that tumor length could be another independent predictive factor. Tumor length of esophageal cancer is considered an important factor related to the degree of peripheral invasiveness of the tumor by researchers (37–39). Song et al. found that tumor length was still an independent predictive factor even in patients with esophageal cancer at the same TNM stages (40). Wang et al. reported that primary tumor length could be used to predictively stratify patients and is a predictive indicator for clinical treatment decisions regarding esophageal cancer treatment (41). Based on previously published literature in terms of predicting response in patients with advanced ESCC, we took these possible predictive factors into consideration and predicted the response in patients after neoadjuvant chemotherapy plus immunotherapy for the first time.

Because the cT stage, cN stage, and tumor length of ESCC obtained on CT were independently associated with the therapeutic response after neoadjuvant chemotherapy plus immunotherapy, the novel nomogram was subsequently developed with the three independent predictors to predict the response. After internal validation of this novel nomogram, the C-index was 0.813, and the calibration curve indicated the perfect accuracy of this model. This model may be a practical and theoretical basis for the clinical pretherapeutic decision-making regarding whether the ESCC patient can benefit from neoadjuvant chemotherapy plus immunotherapy and posttherapeutic follow-up. In clinical practice, clinicians can incorporate cT stage, cN stage, and tumor length based on the pretherapeutic CT features into our nomogram in order to obtain the probability that the patient may be a “responder.” Based on the obtained probability, they can comprehensively make the appropriate treatment decision for the patient. Patients who cannot benefit from neoadjuvant chemotherapy plus immunotherapy even suffer side effects like anemia, decreased white blood cell count, asthenia, vomiting, decreased weight, and rash (12). To improve the quality of life and survival rate of patients with ESCC, we can distinguish the “responders” from “non-responders” before their treatment to recommend that the responders receive neoadjuvant chemotherapy plus immunotherapy while the non-responders do not receive this therapy, avoiding its side effects.

This study had several limitations. On one hand, our research was a single-center retrospective study with a small sample size. Therefore, future work will involve collecting data from multiple centers and large samples to confirm our findings. On the other hand, we did not apply the K-fold cross-validation but the SPSS (version 25, Chicago, IL, USA) for data grouping because the latter has been commonly used in similar research (20). We will compare the utility of K-fold cross-validation to perform the relevant study with the utility of SPSS in the future. In addition, since the conventional thickness of the chest CT scan was 5 mm rather than 1 mm in our hospital, we used 5-mm-thick sections for the retrospective study. We will conduct further research using 1-mm-thick slices to validate the findings in the future. Lastly, the CT images were used to predict the response to neoadjuvant chemotherapy plus immunotherapy rather than to assess the CT nomogram to guide surgery. We will conduct a relevant comprehensive study on the CT nomogram to guide surgery in the future.

In conclusion, we explored the factors for predicting the response of advanced ESCC to neoadjuvant chemotherapy plus immunotherapy and found that cT stage, cN stage, and tumor length are independent predictive factors for prediction. We constructed and validated a new nomogram based on the independent CT predictive factors to predict the response, with good accuracy and reliability. The novel nomogram may be able to help physicians and patients to make appropriate intervention decisions in a timely manner.

## Data availability statement

The raw data supporting the conclusions of this article will be made available by the authors, without undue reservation.

## Ethics statement

The studies involving humans were approved by the Institutional Ethics Committee of the Affiliated Hospital of North Sichuan Medical College. The studies were conducted in accordance with the local legislation and institutional requirements. The participants provided their written informed consent to participate in this study. Written informed consent was obtained from the individual(s) for the publication of any potentially identifiable images or data included in this article.

## Author contributions

WG: Writing – review & editing, Writing – original draft, Visualization, Validation, Methodology, Investigation, Formal Analysis, Data curation. CZ: Writing – original draft, Visualization, Investigation, Data curation. DG: Writing – original draft, Visualization, Validation, Investigation, Formal Analysis. MX: Writing – original draft, Visualization, Investigation, Formal Analysis, Data curation. YG: Writing – review & editing, Supervision, Resources, Methodology, Conceptualization. HZ: Writing – review & editing, Supervision, Software, Methodology, Conceptualization. TC: Writing – review & editing, Writing – original draft, Supervision, Software, Resources, Project administration, Methodology, Funding acquisition, Conceptualization. XZ: Writing – review & editing, Supervision, Software, Resources, Project administration, Methodology, Investigation, Conceptualization.
